# Stable microwave-assisted magnetization switching for nanoscale exchange-coupled composite grain

**DOI:** 10.1186/1556-276X-8-461

**Published:** 2013-11-05

**Authors:** Terumitsu Tanaka, Shota Kashiwagi, Yoshitoki Furomoto, Yuto Otsuka, Kimihide Matsuyama

**Affiliations:** 1 Graduate School of Information Science and Electrical Engineering, Kyushu University, Motooka 744, Nishi-ku, Fukuoka 819-0395, Japan

**Keywords:** Microwave-assisted magnetization reversal, Exchange-coupled composite grain, Micromagnetic simulation

## Abstract

Magnetization mechanisms of nanoscale magnetic grains greatly differ from well-known magnetization mechanisms of micrometer- or millimeter-sized magnetic grains or particles. Magnetization switching mechanisms of nanoscale exchange-coupled composite (ECC) grain in a microwave field was studied using micromagnetic simulation. Magnetization switching involving a strongly damped or precessional oscillation was studied using various strengths of external direct current and microwave fields. These studies imply that the switching behavior of microwave-assisted magnetization switching of the ECC grain can be divided into two groups: stable and unstable regions, similar to the case of the Stoner-Wahlfarth grain. A significant reduction in the switching field was observed in the ECC grain when the magnetization switching involved precessional oscillations similar to the case of the Stoner-Wohlfarth grain. This switching behavior is preferred for the practical applications of microwave-assisted magnetization switching.

## Background

Nanoscale magnetic grains are essential for extending the areal density of hard disk drives. These nanoscale grains are found in hard disk drives, in which the problem of writability still remains to be solved. Energy-assisted magnetic recording schemes [1,2] have already been proposed for solving the writability problems in magnetic recordings. In these recording schemes, microwave-assisted magnetization reversal (MAMR) has recently attracted much attention as an alternative technique for future ultrahigh density recordings. In the case of MAMR, a microwave field is tuned to the ferromagnetic resonance frequency of the recording medium, during which a quasi-direct current (dc) field is also applied, wherein the quasi-dc field is smaller than the switching field in the absence of microwaves. Resonant magnetic precession drives the magnetization over the energy barrier imposed by anisotropy provided that the microwave field amplitude is sufficiently large. Recent experiments [3-6] and simulations [7-13] have demonstrated a reduction in the switching field by applying a large amplitude microwave field with frequencies in the order of gigahertz. To realize ultrahigh density recordings for hard disk drives, magnetic materials with a strong perpendicular magnetic anisotropy (such as *L*1_0_-FePt) are required to overcome thermal fluctuations. However, for magnetization reversal, these materials require a strong magnetic head field and microwave field [14] at extremely high frequencies. This is an issue concerning MAMR that needs to be resolved. Recent micromagnetic analysis has shown that an exchange-coupled composite (ECC) structure [15] with both soft and hard magnetic materials effectively reduces the strengths of dc and microwave fields as well as the optimum microwave frequency for magnetization reversal [16-20].

The analytical treatment for the magnetization of a single magnetic vector under circular microwave fields was discussed [14,21,22]. In these articles, various steady states of precessional magnetization motions were studied by solving the Landau-Lifshitz-Gilbert (LLG) equation. However, there are so far no reports about the steady state of precessional magnetization motions of ECC structured grain. This study presents the magnetization switching behavior of a nanoscale ECC grain using microwave assistance by drawing comparison with the magnetization motions of Stoner-Wohlfarth grain using LLG simulation.

## Methods

**Figure 1:**
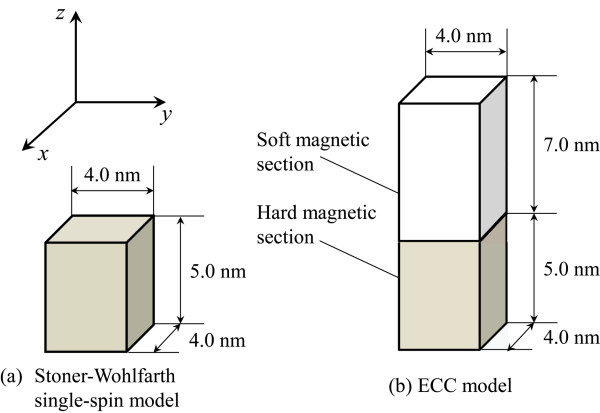
Schematic images of the calculation model (a) Stoner-Wohlfarth grain and (b) ECC grain.

The magnetization mechanisms of the Stoner-Wohlfarth and ECC structured grains were studied by numerically solving the LLG equation. The effective field in the LLG equation was the vector sum of the anisotropy field, magnetostatic field, exchange field, and external dc and microwave fields. Here, the exchange field was not included in the calculation of magnetization behavior for the Stoner-Wohlfarth grain. Rectangular grains were modeled as shown in Figure 1. The grain dimensions are based on recording media of hard disk drives. The thickness of the Stoner-Wohlfarth single spin grain was 5 nm, and those of the soft and hard magnetic sections of the ECC grain were 7 and 5 nm, respectively. The thickness of the soft layer is more than its exchange length (approximately 4 nm). The ECC grain was discretized into 1-nm equilateral cubic prisms, and each prism was assumed to have a single magnetization vector. The uniaxial anisotropy axes of these grains lay in the *z*-direction. The anisotropy field of the Stoner-Wohlfarth grain was 60 kOe, and those of the soft and hard sections for the ECC grain were 10 and 60 kOe, respectively. In the ECC grain, the magnetizations of the soft and hard magnetic sections were ferromagnetically coupled at their interfaces through exchange interaction (1.0 × 10^−6^ erg/cm). All magnetizations were initially arranged in the positive *z*-direction. The dc pulse field, *H*_dc_, was applied in the negative *z*-direction and had a pulse width of 10 ns with a rise/fall time of 1 ns. The circularly polarized microwave field with the strength of *H*_ac_ was also applied in the *x*-*y* plane, where the dc field was constant. These external fields were assumed to be uniformly distributed in the magnetic grains. For all presented results, the exchange stiffness constants for the soft and hard sections were 1.0 × 10^−6^ erg/cm; the dimensionless Gilbert damping constant was 0.05. The saturation magnetization for the Stoner-Wohlfarth grain was 800 emu/cm^3^, and those for the soft and hard sections of the ECC grain were 1,200 and 800 emu/cm^3^, respectively.

## Results and discussion

**Figure 2:**
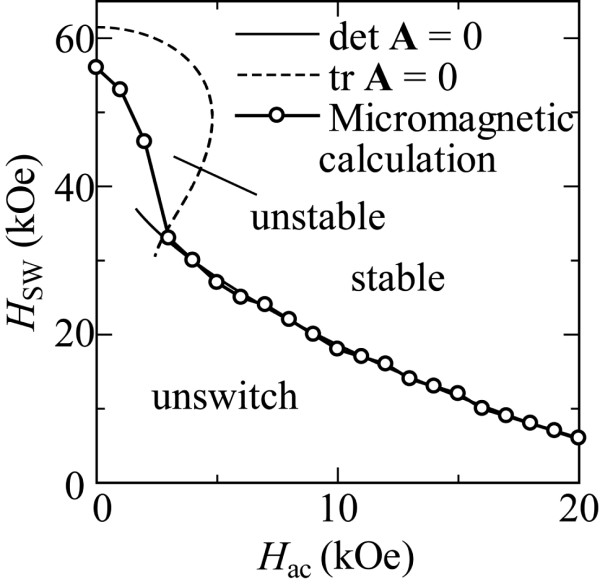
**Curves for detA and trA.** Using 50-GHz microwaves and switching fields of the Stoner-Wohlfarth grain as a function of microwave field strength.

**Figure 3:**
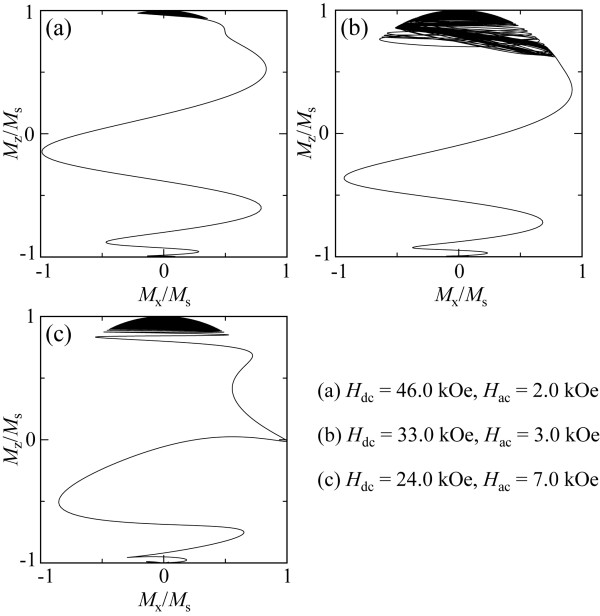
**Trajectories of magnetization projected onto the *x*-*z *plane for the Stoner-Wohlfarth grain. (a)** In the unstable switching region, **(b)** at the boundary of tr**A** = 0, and **(c)** in the stable switching region.

Figure 2 shows the switching field, *H*_SW_, for the Stoner-Wohlfarth grain as a function of *H*_ac_ at 50 GHz. The analytical solutions were obtained by computing the trace and the determinant of the stability matrix expressed by **A**[20]. It is clearly seen that the stable and unstable switching regions observed in the micromagnetic calculation coincide with the region of det**A** = 0 and the region bounded by tr**A** = 0, as derived from Bertotti's analysis. At the boundary of tr**A** *=* 0, *H*_SW_ was confirmed to abruptly increase with decreasing *H*_ac_, which agrees with [14]. Magnetization trajectories during MAMR were calculated using the LLG equation to estimate the switching behavior of the magnetization in the stable and unstable switching regions, which correspond to det**A** = 0 and the region bounded by tr**A** = 0, respectively. Figure 3 presents trajectories of the magnetization vectors, which are projected onto the *x*-*z* plane for the Stoner-Wohlfarth grain in the unstable switching region (a), at the boundary of tr**A** = 0 (b), and in the stable switching region (c). The strengths of the dc and microwave fields are *H*_dc_ = 46 kOe, *H*_ac_ = 2 kOe; *H*_dc_ = 33 kOe, *H*_ac_ = 3 kOe; and *H*_dc_ = 24 kOe, *H*_ac_ = 7 kOe for Figures 3a,b,c, respectively. Large angle precession induced by the microwave field is not observed in the early stage of the magnetization switching in the unstable switching region for condition (a). On the other hand, magnetization switching through a quasiperiodic magnetization mode [21] was observed under condition (b), which was also been demonstrated elsewhere [14]. Magnetization was also confirmed to switch through a pure time-harmonic magnetization mode with no generation of higher-order harmonics (P-mode) in the stable switching region (c).

The theoretical treatment is very useful when analyzing the MAMR process. However, applicable field situations of the treatment are limited [21]. Hence, a numerical integration of the LLG equation is necessary for analyzing MAMR processes under various field situations.

**Figure 4:**
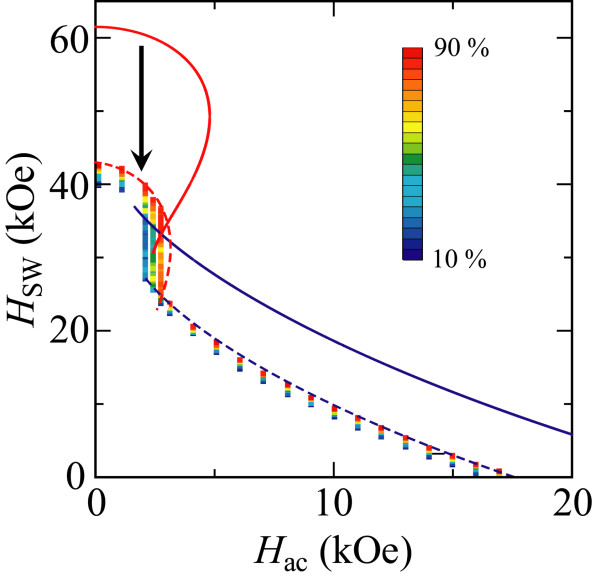
Magnetization switching probability distribution for the Stoner-Wohlfarth grain at 400 K.

Figure 4 shows the probability of magnetization switching events in the Stoner-Wohlfarth grain at the finite temperature *T* = 400 K. The *H*_SW_ in MAMR was theoretically shown to steadily decrease with increasing temperature because of thermal fluctuations [14]. As a result, the stable and unstable switching regions shift toward the lower *H*_SW_ as shown by the broken lines in Figure 4. In the unstable switching region, the switching events were found to widely distribute in *H*_dc_ and *H*_ac_ owing to thermal fluctuations. This implies that larger *H*_dc_ or *H*_ac_ field is necessary for practical applications in magnetic devices utilizing MAMR.

**Figure 5:**
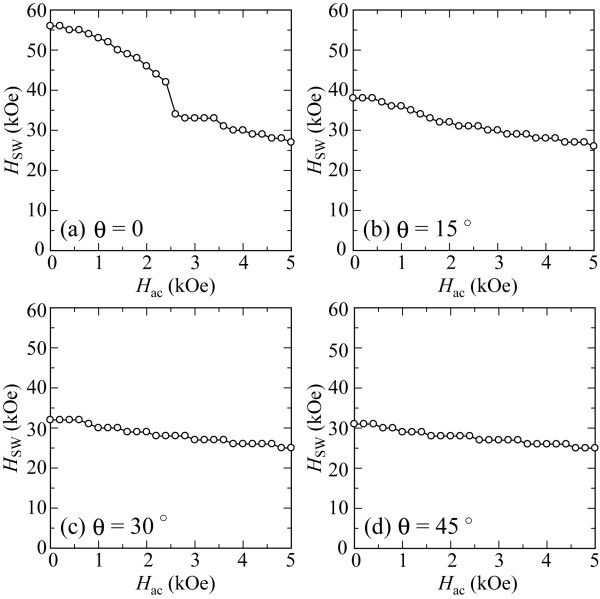
**Switching fields of Stoner-Wohlfarth grain as a parameter of dc field incident angle at 0 K.** With incident angles of **(a)** 0°, **(b)** 15°, **(c)** 30°, and **(d)** 45°.

**Figure 6:**
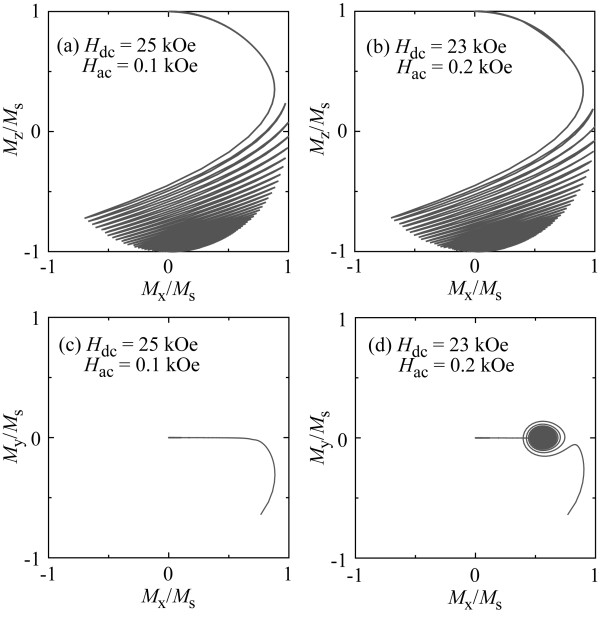
**Trajectories of magnetization projected onto the *x*-*z *plane for Stoner-Wohlfarth grains at 0 K.** They are under the field condition of **(a)***H*_dc_ = 31 kOe, *H*_ac_ = 0.4 kOe and **(b)***H*_dc_ = 30.0 kOe, *H*_ac_ = 0.6 kOe. The field incident angle is 45°. **(c**, **d)** Present trajectories of magnetization projected onto the *x*-*y* plane in the early stage of magnetization switching processes corresponding to (a) and (b)**,** respectively.

Switching fields of the Stoner-Wohlfarth grains are shown in Figure 5 as a parameter of the incident angle of the dc magnetic field at *T* = 0 K. As can be seen in the figure, the strength of *H*_ac_ at which an abrupt change in *H*_SW_ occurs becomes smaller. The change becomes also smaller when the incident angle increases. Considering the magnetization switching process [21,22] under microwave fields, these results are reasonable. These results also imply a shift in the unstable switching region toward smaller *H*_ac_ and *H*_SW_ as well as reduction in the unstable switching region size due to the incident angle. Figure 6 shows trajectories of the magnetization vectors for the Stoner-Wohlfarth grain at an incident angle of 45° when the applied fields are (a) *H*_dc_ *=* 31 kOe, *H*_ac_ *=* 0.4 kOe and (b) *H*_dc_ *=* 30 kOe, *H*_ac_ *=* 0.6 kOe. Figure 6a,b also compares the trajectories of the magnetization projected onto the *x*-*y* plane. The early stages of magnetization switching are shown in Figure 6c,d. These trajectories are apparently different when large-angle magnetization precession is observed at *H*_dc_ = 30 kOe with *H*_ac_ = 0.6 kOe. This qualitatively agrees with the magnetization behaviors shown in Figure 3a,b, which also suggests the shift of the unstable region due to the incident angles.

Although the data is not shown, a great reduction in *H*_SW_ was also confirmed at *T* = 400 K when the incident angle was large. These advantages ensure magnetization switching of high *K*_u_ materials by magnetic fields that are practical in device applications such as hard disk drives.

**Figure 7:**
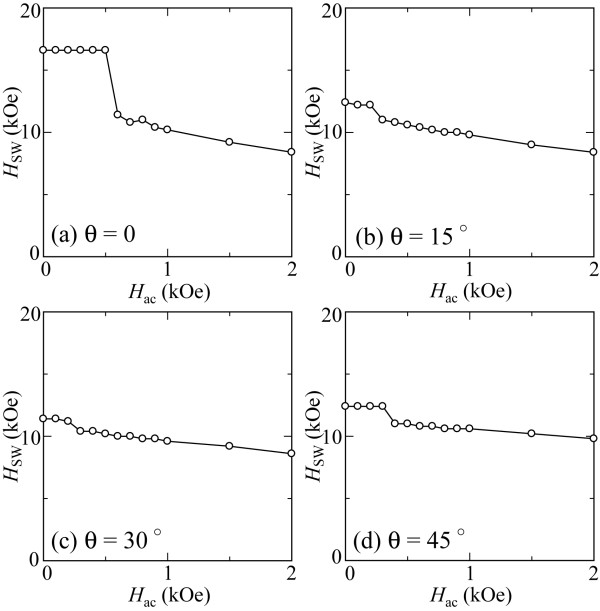
**Switching field of the ECC grain.** The dc field incident angles are **(a)** 0°, **(b)** 15°, **(c)** 30°, and **(d)** 45°.

**Figure 8:**
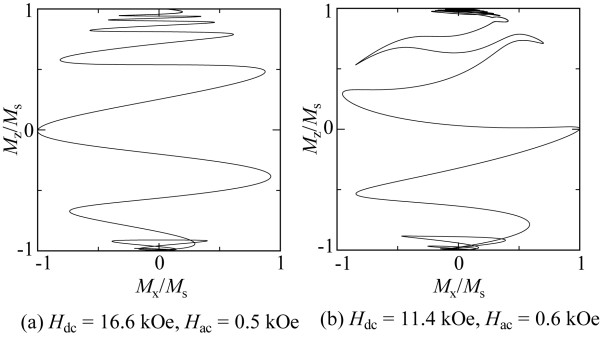
**Trajectories of the magnetization at the top of the hard section for the ECC grain.** Projected onto the *x*-*z* plane under the field conditions **(a)***H*_dc_ = 16.6 kOe, *H*_ac_ = 0.5 kOe and **(b)***H*_dc_ = 11.4 kOe, *H*_ac_ = 0.6 kOe at 0 K. The dc field incident angle is 0°.

During the magnetization switching process of the ECC grain, the magnetization of the soft layer will rotate first under the external field while providing an exchange field to the hard layer to effectively rotate its magnetization, thereby achieving a lower switching field. Soft magnetic layers thicker than their exchange length induce complex incoherent magnetization switching. This means that magnetization mechanisms in the ECC grain cannot be analyzed using the theoretical treatment. Therefore, micromagnetic calculations are required to analyze the stability of magnetization switching in the ECC grain. Figure 7 presents the switching field of the ECC grain with incident angles of 0°, 15°, 30°, and 45° when applying a microwave frequency of 15 GHz. In comparison with the switching field of the Stoner-Wohlfarth grain, a significant reduction in switching fields is obtained in the calculated *H*_ac_ field range. The switching field is minimum when the incident angle is 30°, which is smaller than that for the Stoner-Wohlfarth grain. This tendency is a well-known characteristic in ECC grains in the absence of microwave fields. The abrupt change in *H*_SW_ is also clearly seen at *H*_ac_ = 0.6 kOe when the incident angle is 0°. This implies that the magnetization behavior of the ECC grain can be classified into the three solution regions of the stability matrix, which is similar to the case of Stoner-Wohlfarth grains. The magnetization switching behaviors were also computed to analyze the switching processes in the stable and unstable switching regions for the ECC grain. Figure 8 shows the trajectories of the magnetization at the top of the hard layer projected onto the *x*-*z* plane when the dc and microwave fields are (a) *H*_dc_ = 16.6 kOe, *H*_ac_ = 0.5 kOe and (b) *H*_dc_ = 11.4 kOe, *H*_ac_ = 0.6 kOe at an angle of incidence of 0°. Figure 8a shows magnetization switching induced by large damping in the early stage of the switching process. The magnetization switching process seems to be an unstable switching according to the comparison between theoretical analysis and micromagnetic simulation as shown in Figures 2 and 3, respectively. On the other hand, the precessional oscillation is observed at *H*_dc_ = 11.4 kOe with *H*_ac_ = 0.6 kOe. Magnetization switching involving precessional oscillation was also observed in the stable switching of the Stoner-Wohlfarth grains. This implies that unstable and stable switching occurs under the conditions (a) and (b), respectively, in the ECC grains, indicating that the microwave-assisted switching behavior of the ECC grains qualitatively agrees with the theory predicted by Bertotti [21,22] and micromagnetic simulation by Okamoto [14].

**Figure 9:**
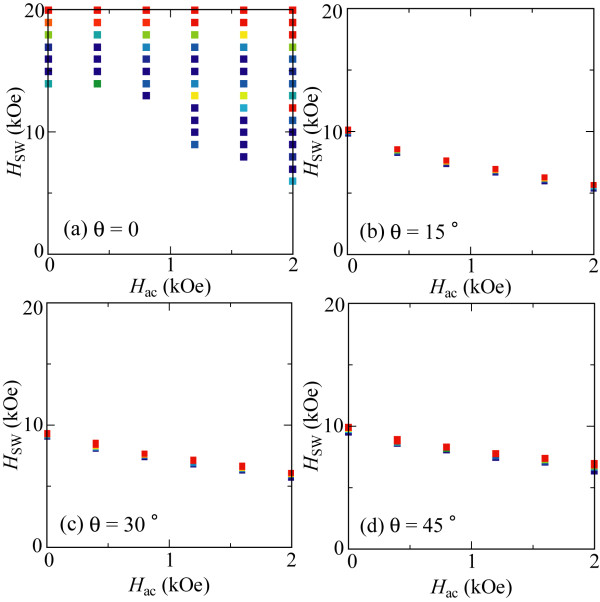
**Magnetization switching probability distribution for the ECC grain at 400 K.** With incident angles of **(a)** 0°, **(b)** 15°, **(c)** 30°, and **(d)** 45°.

Figure 9 shows the probability in magnetization switching events of the ECC grains at the finite temperature *T* = 400 K. Figure 9a,b,c,d is for the incident angles of 0°, 15°, 30°, and 45°, respectively. As concluded from the magnetization behavior shown in Figure 8, the switching probability widely distributes in *H*_dc_ and *H*_ac_ when the incident angle is 0°, which is probably the evidence for unstable switching. On the other hand, the distribution becomes very narrow when the incident angle increases in the same manner as that in Stoner-Wohlfarth grains. This also implies that the reduction in the unstable switching area is due to the incident angles.

## Conclusions

Magnetization switching behavior of a nanoscale ECC grain under microwave assistance has been numerically analyzed by comparing it with that of a Stoner-Wohlfarth grain. The computational simulation indicated that significant switching field reduction due to relatively large microwave field excitation is observed in the ECC grains. Therefore, the magnetization switching in the ECC grain under microwave assistance seems to be divided into two regions of stable and unstable switching depending on applied dc and microwave field strength. Stable switching is more favorable for practical applications when using microwave-assisted magnetization switching in the ECC grain. These results qualitatively agree with the theoretical analysis and the LLG simulation for the Stoner-Wohlfarth grain.

## Competing interests

The authors declare that they have no competing interests.

## Authors’ contributions

TT, SK, YF, and YO carried out the micromagnetic calculation. TT and KM carried out the analysis. All authors read and approve the final manuscript.

## Authors’ information

TT is an assistant professor in ISEE, Kyushu University. His research interests include micromagnetics, magnetic recording, and high frequency magnetic devices. SK received a B.S. degree in Electrical Engineering from Kyushu University in 2013. YF received an M.S. degree in ISEE from Kyushu University in 2013. YO received a B.S. degree in Electrical Engineering from Kyushu University in 2012. KM is a professor in ISEE, Kyushu University. His research interests include magnetic devices.

## References

[B1] Rottmayer RE Batra S Buechel D Challener WA Hohlfeld J Kubota Y Li L Lu B Mihalcea C Mountfield K Pelhos K Peng C Rausch T Seigler MA Weller D Yang X Heat-assisted magnetic recording IEEE Trans Magn 2006 42 2417 2421

[B2] Zhu JG Zhu X Tang Y Microwave assisted magnetic recording IEEE Trans Magn 2008 44 125 131

[B3] Thirion C Wernsdorfr W Mailly D Switching of magnetization by nonlinear resonance studied in single nanoparticles Nature Mater 2003 2 524 527 10.1038/nmat946 12883551

[B4] Moriyama T Cao R Xiao JQ Lu J Wang XR Wen Q Zhang HW Microwave-assisted magnetization switching of Ni_80_Fe_20_ in magnetic tunnel junctions Appl Phys Lett 2007 90 152503 10.1063/1.2720746

[B5] Nozaki Y Ohta M Taharazako S Tateishi K Yoshimura S Matsuyama K Magnetic force microscopy study of microwave-assisted magnetization reversal in submicron-scale ferromagnetic particles Appl Phys Lett 2007 91 082510 10.1063/1.2775047

[B6] Yoshioka T Nozaki T Seki T Shiraishi M Shinjo T Suzuki Y Uehara Y Microwave-assisted magnetization reversal in a perpendicularly magnetized film Appl Phys Express 2010 3 013002 10.1143/APEX.3.013002

[B7] Rivkin K Ketterson JB Magnetization reversal in the anisotropy-dominated regime using time-dependent magnetic fields Appl Phys Lett 2006 89 252507 10.1063/1.2405855

[B8] Nozaki Y Matsuyama K Numerical study for ballistic switching of magnetization in single domain particle triggered by a ferromagnetic resonance within a relaxation time limit J Appl Phys 2006 100 053911 10.1063/1.2338128

[B9] Okamoto S Kikuchi N Kitakami O Magnetization switching behavior with microwave assistance Appl Phys Lett 2008 93 102506 10.1063/1.2977474

[B10] Scholz W Batra S Micromagnetic modeling of ferromagnetic resonance assisted switching J Appl Phys 2008 103 07F539 10.1063/1.2838332

[B11] Gao KZ Benakli M Energy surface model and dynamic switching under alternating field at microwave frequency Appl Phys Lett 2009 94 102506 10.1063/1.3097229

[B12] Wang X Gao KZ Hohlfeld J Seigler M Switching field distribution and transition width in energy assisted magnetic recording Appl Phys Lett 2010 97 102502 10.1063/1.3486167

[B13] Tanaka T Kato A Furomoto Y Md Nor AF Kanai Y Matsuyama K Microwave-assisted magnetic recording simulation on exchange-coupled composite medium J Appl Phys 2012 111 07B711 10.1063/1.3678450

[B14] Okamoto S Igarashi I Kikuchi N Kitakami O Microwave assisted switching mechanism and its stable switching limit J Appl Phys 2010 107 123914 10.1063/1.3436570

[B15] Victora RH Shen X Composite media for perpendicular magnetic recording IEEE Trans Magn 2005 41 537 542

[B16] Bashir MA Schrefl T Dean J Goncharov A Hrkac G Bance S Allwood D Suess D Microwave-assisted magnetization reversal in exchange spring media IEEE Trans Magn 2008 44 3519 3522

[B17] Li S Livshitz B Bertram HN Schabes M Schrefl T Fullerton EE Lomakin V Microwave assisted magnetization reversal in composite media Appl Phys Lett 2009 94 202509 10.1063/1.3133354

[B18] Igarashi M Suzuki Y Miyamoto H Maruyama Y Shiroishi Y Mechanism of microwave assisted magnetic switching J Appl Phys 2009 105 07B907 10.1063/1.3075850

[B19] Li H Hou F Li P Yang X Influences of switching field rise time on microwave-assisted magnetization reversal IEEE Trans Magn 2011 47 355 358

[B20] Tanaka T Narita N Kato A Nozaki Y Hong YK Matsuyama K Micromagnetic study of microwave-assisted magnetization reversals of exchange-coupled composite nanopillars IEEE Trans Magn 2013 49 562 566

[B21] Bertotti G Serpico C Mayergoyz D Nonlinear magnetization dynamics under circularly polarized field Phys Rev Lett 2001 86 724 727 10.1103/PhysRevLett.86.724 11177922

[B22] Bertotti G Mayergoyz ID Serpico C d’Aquino M Bonin R Nonlinear-dynamical-system approach to microwave-assisted magnetization dynamics J Appl Phys 2009 105 07B712 10.1063/1.3072075

